# Supplementary material for: Red-edge position of habitable exoplanets around M-dwarfs
Source: Sci Rep. 2017 Aug 8;7:7561. doi: 10.1038/s41598-017-07948-5 (PMC5548919; doi:10.1038/s41598-017-07948-5)

Supplementary Information

Red-edge position of habitable exoplanets around M-dwarfs

Kenji Takizawa[1,2], Jun Minagawa[2], Motohide Tamura[1,3,4], Nobuhiko Kusakabe[1,4], *Norio Narita[1,3,4]

[1] Astrobiology Center, National Institutes of Natural Sciences, 2-21-1 Osawa, Mitaka, Tokyo 181-8588, Japan

[2] National Institute for Basic Biology, National Institutes of Natural Sciences, 38 Nishigonaka, Myodaiji, Okazaki, Aichi
444-8585, Japan

[3] Department of Astronomy, The University of Tokyo, 7-3-1 Hongo, Bunkyo-ku, Tokyo 113-0033, Japan

[4] National Astronomical Observatory of Japan, National Institutes of Natural Sciences, 2-21-1 Osawa, Mitaka, Tokyo
181-8588, Japan

*Corresponding author: Norio Narita (norio.narita@nao.ac.jp)

Supplementary Table 1 | Photon flux density at the top of atmosphere (TOA), land surface, and under water at different depths. The peak wavelength and visible-light (400−700 nm) intensity were compared among present Earth, Earth 2.7 billion years ago, and a hypothetical habitable planet around AD Leo.

		Photon Flux Density					
		peak wavelength (nm)			400−700 nm (μmol $m^{-2}s^{-1}$)		
		Sun/Earth	(2.7 Gya)	AD Leo	Sun/Earth	(2.7 Gya)	AD Leo
TOA		583	(583)	1004	2415	(1958)	350
Surface		669	(674)	1005	1738	(1474)	266
underwater	0.01	669	(608)	884	1731	(1468)	264
(m)	0.1	669	(608)	809	1670	(1417)	251
	1	531	(549)	656	1238	(1046)	160
	10	495	(498)	539	401	(333)	35
	100	495	(498)	494	1.1	(0.9)	0.08

Supplementary Figure 1 | EFD and PFD spectra for Earth (panels a and c) and a hypothetical habitable planet around AD Leo (panels b and d). Gray lines show the spectra obtained at the top of the atmosphere. Black lines show the spectra at the land surface passing through a 1.5 air-mass atmosphere (the solar zenith angle is 48.2°). The underwater EFDs and PFDs at a depth of 0.1, 1, and 10 m are shown using red, green, and blue lines, respectively.

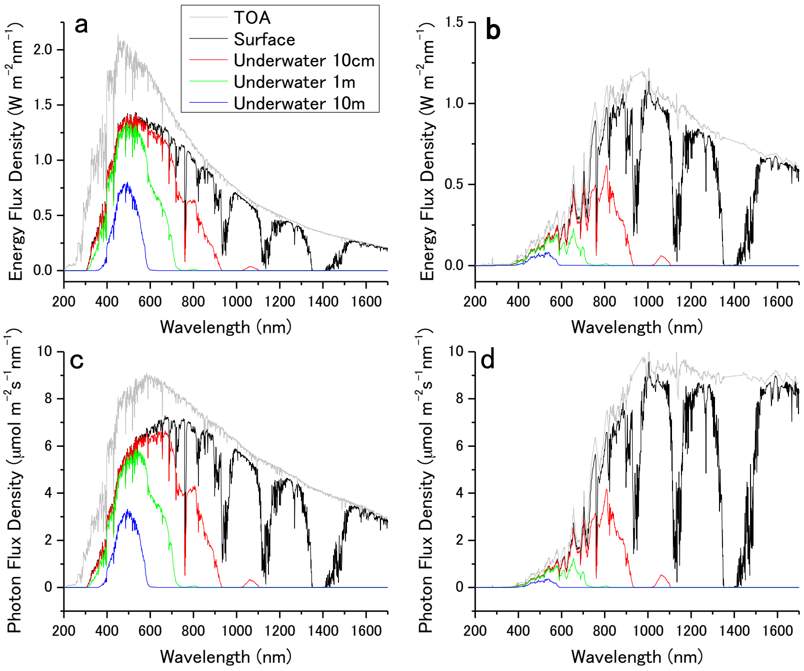

Supplementary Figure 2 | Changes in integrated PFD due to changing light absorption band position and bandwidth. Integrated PFD for solar radiation (panels a and c) and AD Leo's radiation (panels b and d) at the planet surface (black lines) and underwater at depths of 0.1 (red lines), 1 (green lines), and 10 m (blue lines) were calculated from PFD spectra (Supplementary Figure 1, panels c and d). In panels a and b, photon fluxes were integrated over a bandwidth of 300 nm. The horizontal axis indicates the reaction center absorption wavelength, which is the longest wavelength absorbed by light-harvesting pigments. In panels c and d, photon fluxes are integrated between 400 nm and the excitation wavelength of the reaction center, which is shown in the horizontal axis.

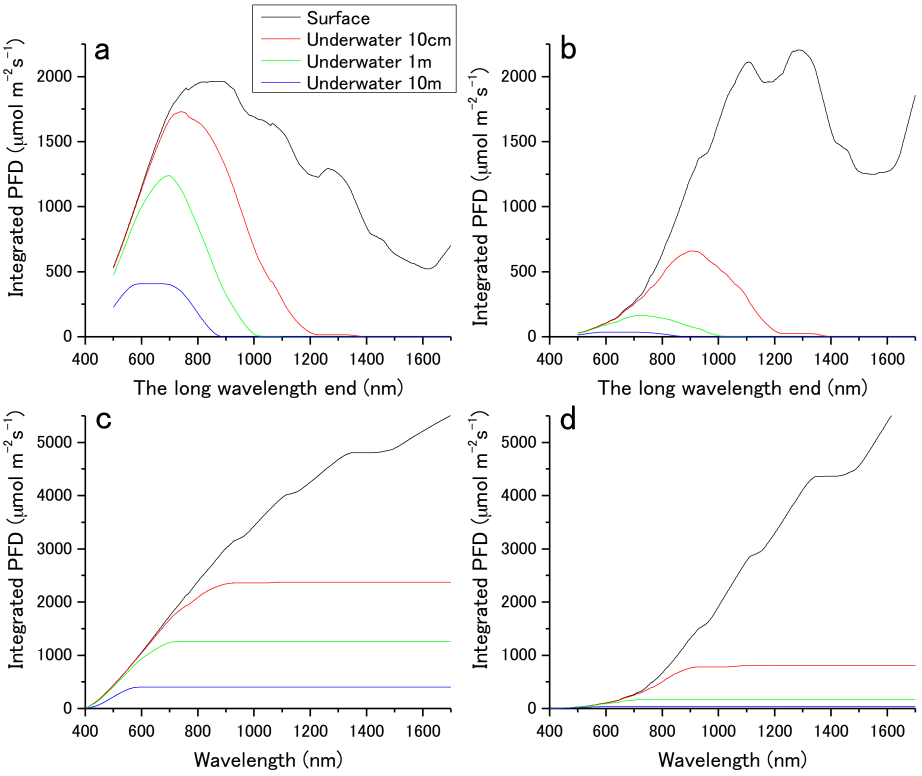

Supplementary Figure 3 | Changes in the energy input for the reaction centers (RCs) due to changing light absorption wavelengths. The excitation energy obtained by the reaction centers is calculated for different light conditions: on land surface (black lines) and underwater at depths of 0.1 (red lines), 1 (green lines), and 10 m (blue lines). Panels a and c show the energy input from solar radiation. Panels b and d show the energy input from AD Leo's radiation. In panels a and b, the light absorption bandwidth is fixed at 300 nm and the excitation wavelength of the reaction center is changed from 400 to 1,500 nm, as shown on the x-axis. In panels c and d, the light absorption bandwidth is increased from 0 to 1,100 nm as the excitation wavelength of the reaction center increases from 400 to 1,500 nm.

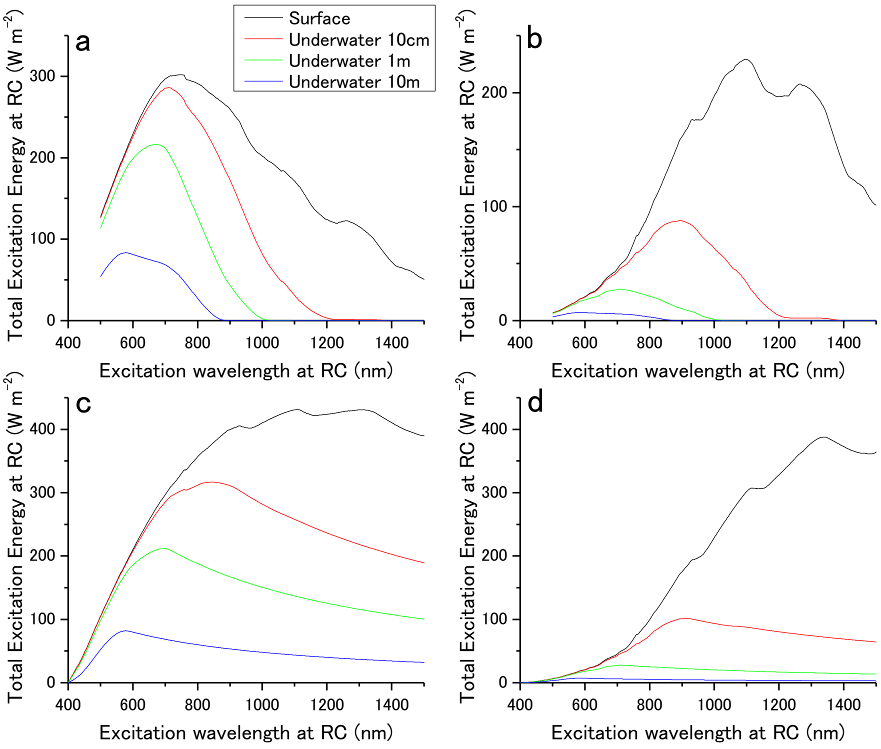

Supplementary Figure 4 | Energy transfer sequences for hypothetical two- and three-photon reactions. A sharp rise in the reverse sawtooth wave indicates the energy input by photochemical reactions; a stepwise decrease indicates energy loss by subsequent energy transductions (i.e., electron transport). Black squares indicate the two-photon reaction that receives two photons at 700 nm (2 x 1.77 V) and stores 1.23 V of chemical energy (35% efficiency). Each photosystem loses 1.16 V during energy transfer. Blue triangles indicate the three-photon reaction that receives three photons at 1,050 nm (3 x 1.18 V) and stores 1.23 V of chemical energy (35% efficiency). Each photosystem loses 0.77 V during energy transfer. Red circles indicate the three-photon reaction that receives three photons at 791 nm (3 x 1.57 eV) and stores 1.23 V of chemical energy (26% efficiency). Each photosystem loses 1.16 V during energy transfer. If the three-photon reaction evolves from the two-photon reactions (black squares) and gains minor improvements in energy storage during electron transfer, the total electron efficiency of the three-photon reaction will be between 35% (blue triangles) and 26% (red circles).

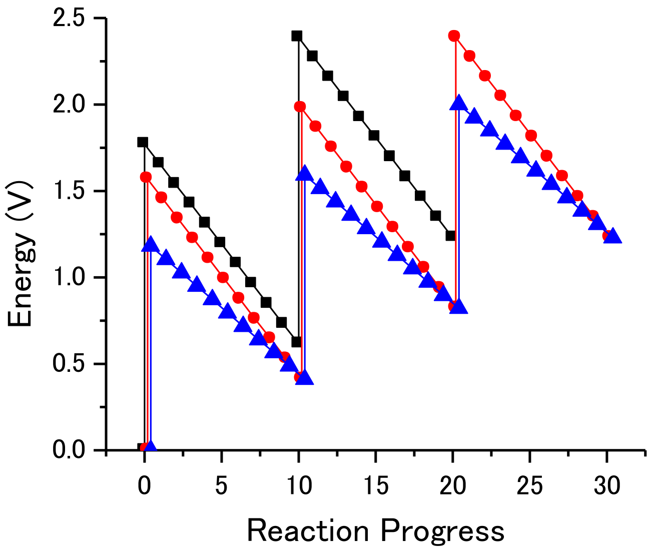

Supplementary Figure 5 | O_2 production rates for multiple-photon reactions. The O_2 production rates under solar (panel a) and AD Leo's (panel b) radiation are calculated. The PFDs obtained for the land surface were integrated between 400 nm and the excitation wavelength of the reaction center (as in Supplementary Figure 2, panels c and d) and converted to the O_2 production rate for two-, three-, or four-photon reactions. Black dots on black lines (two-photon reaction) show the O_2 production rate for Earth-type photosynthesis, which absorbs radiation in the range of 400–700 nm. The red and blue lines show the O_2 production rates for three- and four-photon reactions, respectively. The thick lines indicate the energetically appropriate wavelength range.

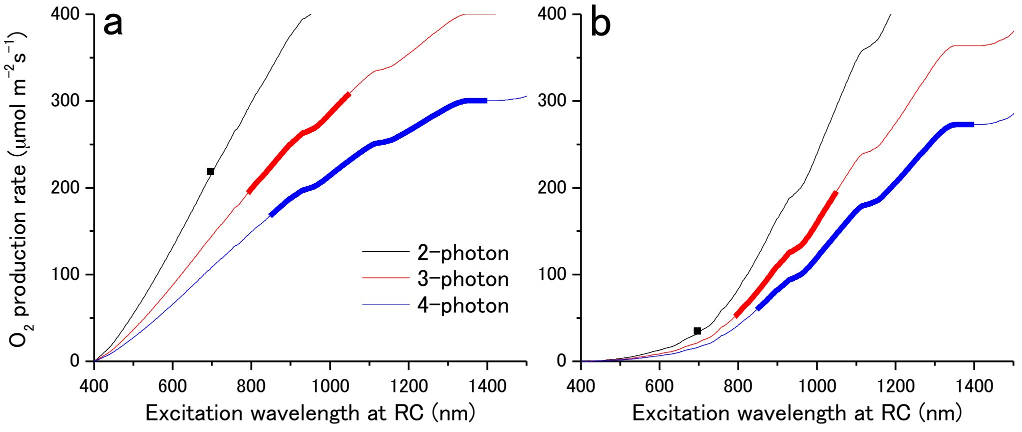

Supplement: Supplementary file 1 — Supplementary information [file 41598_2017_7948_MOESM1_ESM.pdf]
